# Low Dose of Valproate Improves Motor Function after Traumatic Brain Injury

**DOI:** 10.1155/2014/980657

**Published:** 2014-02-06

**Authors:** Yu-Ting Tai, Wen-Yuan Lee, Fei-Peng Lee, Tien-Jen Lin, Chia-Lin Shih, Jia-Yi Wang, Wen-Ta Chiu, Kuo-Sheng Hung

**Affiliations:** ^1^Department of Anesthesiology, Taipei Medical University-Wan Fang Hospital, Taipei Medical University, Taipei 116, Taiwan; ^2^Department of Neurosurgery, China Medical University Hospital, China Medical University, Taipei Branch, Taipei, Taiwan; ^3^Department of Otolaryngology, Clinical Research Center, School of Medicine, Wan Fang Hospital, Taipei Medical University, Taipei, Taiwan; ^4^Department of Neurosurgery, Clinical Research Center, Graduate Institute of Injury Prevention and Control, Wan Fang Hospital, Taipei Medical University, No. 111, Section 3, Hsing-Long Road, Taipei 116, Taiwan; ^5^Graduate Institute of Biomedical Sciences, College of Medicine, Taipei Medical University, Taipei, Taiwan

## Abstract

*Background.* Traumatic brain injuries (TBIs) are a major health care problem worldwide. Approximately 1.5 million new TBI cases occur annually in the United States, with mortality rates ranging between 35% and 40% in severe patients. Despite the incidence of these injuries and their substantial socioeconomic implications, no specific pharmacological intervention is available for clinical use. Several studies have indicated that 300 mg/kg or 400 mg/kg of valproate (VPA) exhibits neuroprotective effects in animal models. However, humans cannot tolerate high doses of VPA. This study aims to investigate whether 30 mg/kg of VPA administered to rats affects TBIs. *Methods.* We used a rat model to test the effects of 30 mg/kg of VPA on TBIs. Molecular identifications for histone acetylation and phosphorylation of cAMP response element-binding protein (CREB) and phosphorylated extracellular signal regulated kinase (ERK) were performed. *Results.* The results indicated that treating adult rats with VPA after TBIs significantly decreased the contusion volume and recovery of contusion-related skilled forelimb reaching deficits. Applying VPA also increased histone acetylation, p-ERK, and p-CREB expression in the brain. Furthermore, applying VPA reduced inflammation, glial fibrillary acidic protein activation, and apoptosis. *Conclusion.* This study found that 30 mg/kg of VPA assists in treating TBIs in rat models.

## 1. Introduction

Traumatic brain injury (TBI) is a major clinical problem that causes substantial mortality rates and a broad spectrum of mental disorders. These complex pathological conditions are characterized by blood brain barrier (BBB) leakage, excessive release of excitatory neurotransmitters, axonal and dendritic disruptions, neuroinflammation, and cell death [1–4]. Given the complexity of brain responses to trauma and the lack of an ideal drug for treating TBIs, the National Institutes of Health TBI working group recommended either a combination of therapies or evaluating agents that act on multiple mechanisms as TBI treatment options [5].

Valproate [2-propylpentanoic acid] (VPA) is one of the most commonly used antiepileptic medications. It has been shown to reduce the neuronal damage associated with epileptic activity. After a TBI, VPA has been shown to be effective in treating posttraumatic seizures [6]. The antiepileptic activity of VPA results from a combination of its influences on several targets in the central nervous system, including inhibiting gamma amino butyric acid transamination, reducing N-methyl D-aspartate excitotoxic amino acid (NMDA)-mediated neuronal excitation, inhibiting histone deacetylases (HDACs) and glycogen synthase kinase (GSK)-3, and blocking voltage-gated sodium and T-type calcium channels [7]. Studies have found that 300 mg/kg or 400 mg/kg of VPA exhibits neuroprotective effects in animals [8–10]. However, humans cannot tolerate such large doses, which might be toxic [11]. In this study, we administered 30 mg/kg of VPA to rats to test whether this dose is effective in treating TBIs, given that 30 mg/kg of VPA is acceptable for clinical practice.

## 2. Methods

### 2.1. Materials

The experimental procedures used in this study conformed to the guidelines approved by the institutional animal care committee at Wan Fang Hospital. Adult male Sprague-Dawley rats (250 to 300 g) were obtained from BioLASCO Taiwan Co., Ltd. The rats were randomly divided into 3 groups: (1) the TBI + VPA (VPA-treated TBI) group, (2) the TBI (vehicle-treated TBI) group, and (3) the sham (sham-operated control) group. They were housed in a temperature-controlled animal room (24°C to 25°C) and exposed to a 12 h light-dark cycle. Standard laboratory rat chow and tap water were available ad libitum. Acetylated histone H2A, acetylated histone H2B, and acetylated histone H3 antibodies were obtained from Millipore (Billerica, MA, USA). Phospho-ERK42/44 and phospho-CREB antibodies were purchased from Cell Signaling Technology (Danvers, MA, USA). Anti-beta actin and glial fibrillary acidic protein (GFAP) antibodies were obtained from Sigma-Aldrich Biotechnology (Saint Louis, MO, USA). Apoptosis detection kits, including a terminal deoxynucleotidyl transferase-mediated-UTP-biotin nick end labeling (TUNEL) assay and 4′,6-diamidino-2-phenylindole (DAPI) staining kits, were purchased from Oncogene Research Products (Boston, MA, USA).

### 2.2. Controlled Cortical Impact TBI Model

Surgical anesthesia was induced by intraperitoneally (IP) administering ketamine (90 mg/kg) and xylazine (10 mg/kg). After anesthesia, the animals were secured in a stereotaxic frame and mechanically ventilated. A cortical contusion was produced on the exposed cortex by using a controlled impactor device, the TBI-0200 TBI Model system (Precision Systems and Instrumentation). The scalp and epicranial aponeuroses were retracted, and a 3 mm diameter circular craniotomy was performed with a burr drill, lateral to the midsagittal suture (contralateral to the preferred limb), with its center at the following coordinates: AP = 1 mm and ML = ±2.5 mm from the bregma. The impacting shaft was extended, and the impact tip was centered and lowered over the craniotomy site until it touched the dura mater. The rod was then retracted and the impact tip was advanced to produce a brain injury of moderate severity to a rat (tip diameter, 3 mm; cortical contusion depth, 2 mm; impact velocity, 4 m/s).

### 2.3. Drug Preparation, Administration, and Grouping

VPA (Depakine lyophilized injection, Sanofi-Aventis) was dissolved in 0.9% sterile saline at a concentration of 100 mg/mL (based on the salt weight). Animals received either an IP VPA injection of 30 mg/kg/d or a vehicle (0.9% sterile saline) 30 min after the TBI from day 0 to day 6. Skilled forelimb reaching tests (*n* = 8 per group), contusion volume measurements (*n* = 5 per group), and immunohistochemistry studies (*n* = 3 per group) were then conducted. Some animals received either a single IP VPA injection of 100 mg/kg, 200 mg/kg, or 400 mg/kg or a vehicle (0.9% sterile saline) 30 min after TBI to perform western blot analyses (*n* = 3 per group).

### 2.4. Training or Skilled Forelimb Reaching Test

Animals were trained to criterion in the skilled forelimb reaching task and their performances were assessed. The rats were randomly allocated to 3 groups (*n* = 8 per group): (1) the TBI + VPA (VPA-treated TBI) group, (2) the TBI (vehicle-treated TBI) group, and (3) the sham (sham-operated control) group. Each animal was tested to assess their skilled forelimb reaching task performance on the first postoperative day and then daily (Monday to Friday) for 6 weeks. On days when VPA or the vehicle was administered (i.e., days 0 to 6), behavior testing was performed before drug injection. Skilled forelimb reaching was tested as described by [12]. Before surgery (contralateral to the preferred limb craniotomy), the baseline performance (defined as the average of the last 3 preoperative testing sessions) of each rat was established. Success was defined as an animal grasping the pellet on its first attempt and placing it into its mouth (this is termed “first reach success”). Each testing session consisted of 20 reaching opportunities, using the preferred forelimb. Attempts using the nonpreferred forelimb were not included in the analyses. The preoperative criterion was at least 16 successes in 20 attempts for 3 consecutive days. A maximal time limit of 5 min per testing session was set.

### 2.5. Triphenyltetrazolium Chloride Staining

2,3,5-triphenyltetrazolium chloride (TCC) staining was used to assess the lesion size by comparing various neuronal tissue viabilities. The excised brain was sliced into 2 mm thick sections and incubated in a 1% TTC solution for 30 min at 37°C. In viable neuronal tissue, dehydrogenase enzymes converted TTC into a red pigment that stained the tissue dark red.

### 2.6. Contusion Volume Measurement

To describe the entire contusion volume for further analysis, the contusion volume areas were specifically analyzed. Images of all sections were captured using a digital camera (Nikon Coolpix 990) and analyzed using ImageJ software (public domain program developed at the National Institutes of Health, Bethesda, MD, USA).

### 2.7. Western Blotting

Rats received either a single IP VPA injection of 30 mg/kg, 100 mg/kg, 200 mg/kg, or 400 mg/kg or a vehicle (0.9% sterile saline) 30 min after the TBI (*n* = 3 per group). Ipsilateral frontal cortical segments were obtained 24 h after the VPA or vehicle injection and homogenized. Brain tissue extracts were sonicated (five pulses per second) by using a Sonics Vibra-Cell sonicator (Sonics & Materials, Inc., Newtown, CT, USA) and a 0.4 mm diameter probe. The amount of protein in each sample was determined using a Bradford assay with bovine serum albumin as the standard. Equal amounts of protein were loaded, electrophoresed, and transferred to Immobilon-P membranes (Millipore, Billerica, MA, USA) by using the NOVEX X-Cell II system (Invitrogen, Burlingame, CA, USA). Membranes were then washed and incubated with antibodies at room temperature. A chemiluminescence system was used to detect immunoreactivity.

### 2.8. Immunohistochemistry

To examine the effects of VPA after a TBI, animals were operated on and their brains were sectioned for post-TBI histological evaluation (*n* = 3 per group). Three or 7 days after the TBI, rats were sacrificed for H&E staining, immunocytochemical GFAP analyses, or TUNEL staining. Animals were deeply anesthetized with an IP injection of 150 mg/kg of pentobarbital and perfused through the left ventricle with phosphate-buffered saline, followed by cold 4% paraformaldehyde in 0.15 M sodium phosphate buffer, pH 7.4. The brains were immediately removed, postfixed for 8 h in the same fixative at 4°C, and cryoprotected for 2 to 3 days in 15% and 30% sucrose. The brains were frozen in powdered dry ice and stored at −80°C until required and 30 *μ*m coronal sections were then cut using a freezing, sliding microtome. The sections were prepared for either immunostaining or apoptosis staining.

### 2.9. Statistics

A one-way ANOVA was used to analyze brain contusion lesion size. Skilled forelimb reaching was independently analyzed using a repeated-measures one-way ANOVA with Tukey post hoc analysis for the treatment effect. Data were considered significant at *P* < .05 and are presented as the mean ± the standard error of the mean (SEM).

## 3. Results

### 3.1. VPA Administered after a TBI Reduces Brain Contusion Volume

**Figure 1:**
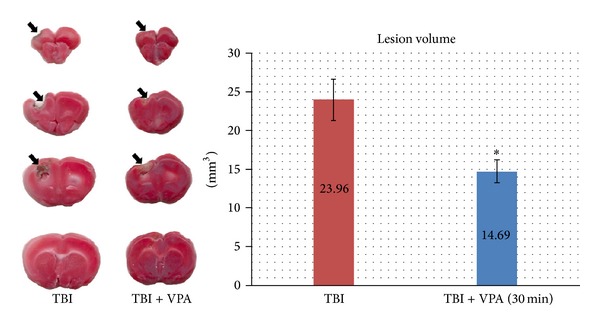
Postinjury administration of 30 mg/kg/day of VPA for 7 days reduces contusion volume. Representative photographs of the brains of a vehicle- and VPA-treated animal. Animals were killed 7 days after injury. Quantification of the volume of cortical contusion revealed that VPA significantly reduced brain contusion volume (mean: 23.96 versus 14.19 mm^3^). Rostral-caudal extent of the damage detected in the injured cortex from the vehicle- and VPA-treated animals. (Data are presented as the mean ± SEM, significant difference by 1-way ANOVA. *P = .010, *n* = 5 per group.)

We investigated continuous IP treatment with 30 mg/kg/d of VPA that began 30 min after the TBI and lasted for 7 days. Seven days after the TBI, VPA and vehicle-injected animals were euthanized and their brains were removed to determine the contusion volume. As shown in Figure 1, the animals treated with 30 mg/kg/d of VPA for 7 days exhibited less cortical tissue loss than the vehicle-treated animals did. Quantification of this tissue loss revealed that 30 mg/kg/d of VPA produced significantly less tissue loss than vehicle injections did (*P* = .01; Figure 1).

### 3.2. VPA Improves Skilled Motor Function after a TBI

**Figure 2:**
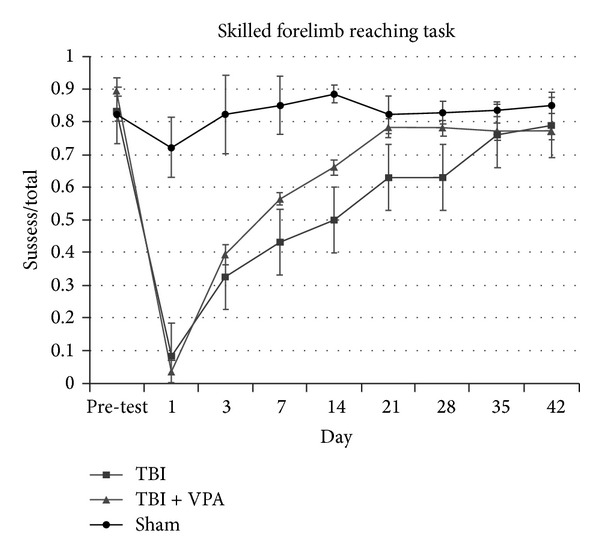
Postinjury treatment of VPA improves skilled forelimb reaching task. All groups had a baseline performance without a significant difference. After TBI, the vehicle- and VPA-treated groups showed marked deficits in successfully obtaining pellets with the TBI-impaired limb, with no significant difference between groups until 7 days after injury. Animals that received VPA treatment, 30 mg/kg/day for 7 days began to exhibit improvements in the pellet reaching success rate at 7 days after TBI and showed a significant difference starting from 14 to 28 days after treatment, compared to TBI with vehicle-only animals (14 days, *P* = .013; 21 days, *P* = .005; 28 days, *P* = .005). Data are presented as the mean ± SEM, significant difference by 1-way ANOVA with Tukey post hoc analysis.

Skilled forelimb reaching, which requires fine digit movement and intact motor and sensory neural pathways, was analyzed using the single pellet retrieval task [13]. Before TBI surgery, animals in all groups showed good skilled reaching without significant difference in performance. Three days after the TBI, all animals exhibited significant deficits in obtaining pellets with the TBI-impaired limb (Figure 2). However, animals that received 30 mg/kg/d of VPA for 7 days began to increase their pellet reaching success rate 7 days after the TBI and showed significant differences to the TBI with vehicle-only animals 14 to 28 days after treatment (14 d, *P* = .013; 21 d, *P* = .005; 28 d, *P* = .005; Figure 2). A repeated-measures one-way ANOVA revealed that a significant treatment effect occurred (*P* = .038) after 6 weeks of testing. The results demonstrated that postinjury VPA treatment improves skilled motor function after TBIs.

### 3.3. Systemic VPA Administration Increases H3 Histone Acetylation, p-ERK, and p-CREB in the Brain

**Figure 3:**
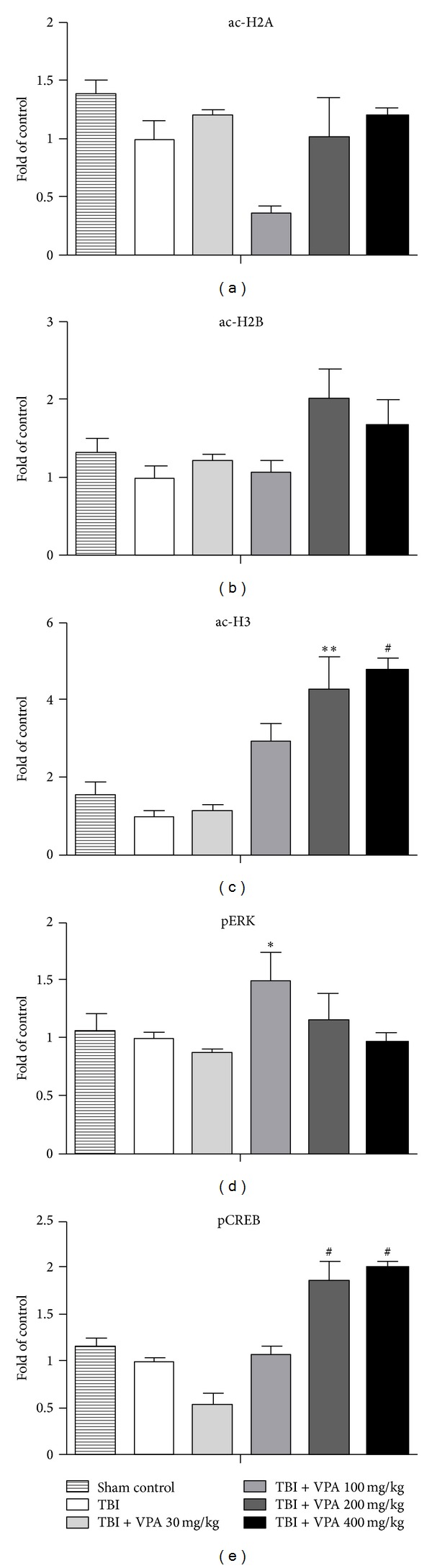
Systemic VPA administration increases H3 histone acetylation, p-ERK, and p-CREB in brain. The statistical analysis of the quantification of western blots (c) showed that systemic VPA administration (*n* = 3 per group) significantly increases histone H3 acetylation with the dosage of 200 mg/Kg (*P* = .0087) and 400 mg/Kg (*P* = .006) 1 day after treatment compared to the vehicle-treated controls. No significant changes were detected when the levels of acetyl-H2A and acetyl-H2B histone antibodies were evaluated (a and b). Significant increase of p-ERK expression was noted 1 day after 100 mg/kg VPA treatment compared to vehicle-treated controls (d), (*P* = .0488). Also increased expression of p-CREB was noted with the dosage of 200 mg/Kg (*P* < .0001) and 400 mg/Kg (*P* < .0001) 1 day after VPA treatment compared to the vehicle-treated controls (e). These results demonstrate that systemic VPA administration increases H3 histone acetylation, p-ERK, and p-CREB expressions in the brain.

VPA is an HDAC inhibitor and therefore preserves the acetylation of histones [10]. To examine the relationship between systemic VPA administration and histone acetylation in the brain, western blots were performed with acetyl-H2A, acetyl-H2B, and acetyl-H3 histone antibodies. Brain protein extracts were prepared for the western blots. The representative western blots (Figure 3(c)) indicated that 200 mg/kg (*P* = .0087) and 400 mg/kg (*P* = .006) of VPA significantly increased histone H3 acetylation 1 day after VPA treatment, compared with the vehicle-treated controls. No significant changes were detected when the levels of acetyl-H2A and acetyl-H2B histone antibodies were evaluated (Figures 3(a) and 3(b)).

As well as inhibiting HDAC, VPA activates the ERK and CREB pathways [7, 14]. To examine this effect of VPA administration, the p-ERK and p-CREB levels were evaluated for 4 VPA doses (30 mg/kg, 100 mg/kg, 200 mg/kg, and 400 mg/kg). A significant increase in phospho-ERK expression occurred 1 day after the 100 mg/kg VPA treatment, compared with the vehicle-treated controls (*P* = .0488; Figure 3(d)). The 200 mg/kg (*P* < .0001) and 400 mg/kg (*P* < .0001) doses resulted in increased p-CREB expression 1 day after VPA treatment, compared with the vehicle-treated controls (Figure 3(e)). These results demonstrated that systemic VPA administration increases H3 histone acetylation and p-ERK and p-CREB expression in the brain.

### 3.4. VPA Administered after a TBI Reduces Inflammation, GFAP Expression, and Apoptosis

**Figure 4:**
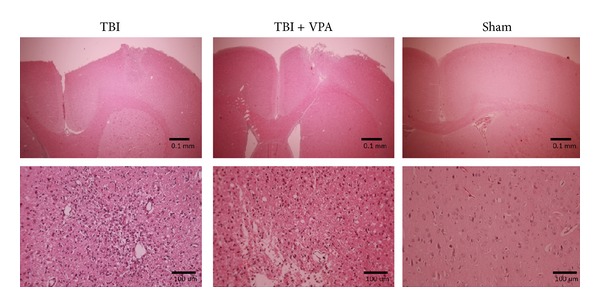
VPA administered after TBI reduces inflammation. H&E staining shows pathological change 7 days after TBI; VPA treatment (30 mg/kg/day for 7 days) was able to reduce the number of inflammatory cells compared to the vehicle alone.

**Figure 5:**
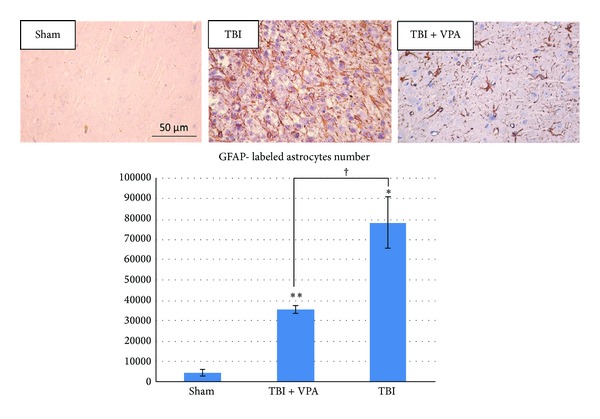
Postinjury administration of 30 mg/kg/day VPA for 7 days reduces GFAP expression. Strong immunostaining of GFAP was demonstrated 7 days after TBI, but these activations were significantly attenuated in the VPA-treated group. (Data are presented as the mean ± SEM, significant difference by 1-way ANOVA followed by Dunnett's post hoc test. ^†^ *P* = .005, *n* = 3 per group.)

**Figure 6:**
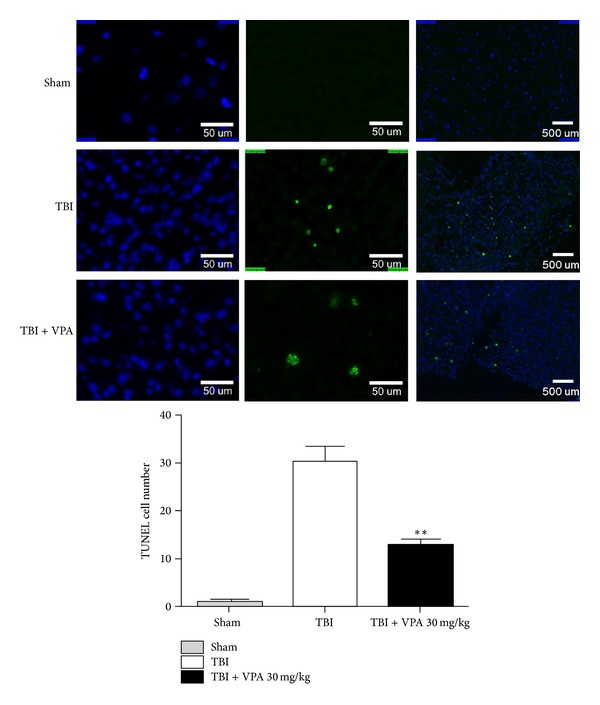
VPA attenuates apoptosis after TBI. TUNEL-positive cells with green fluorescence were easily demonstrated in the TBI group. After VPA treatment (30 mg/kg/day for 7 days), the apoptotic cells significantly decreased. (Data are presented as the mean ± SEM, significant difference by 1-way ANOVA followed by Dunnet's post hoc test. **P = .0072, *n* = 3 per group.)

Figure 4 shows the H&E stain of the pathological change that occurred 7 days after a TBI. The figure shows that 30 mg/kg/d of VPA treatment for 7 days reduced inflammatory cells to a greater extent than the vehicle did. Astrocyte activation contributes to the inflammatory response after a TBI. We investigated the expression of GFAP—a marker of activated astrocytes and potential TBI severity grading. Strong GFAP immunostaining occurred 7 days after the TBI, but the VPA-treated group exhibited significantly weaker activations (Figure 5, *P* = .005, *n* = 3 per group). These results indicated that postinjury treatment with VPA (30 mg/kg/d for 7 d) attenuated the activation of astrocytes and inflammation after the TBI. Additionally, after 7 days of 30 mg/kg/d of VPA treatment, the number of apoptotic cells significantly decreased (Figure 6, *P* = .0072, *n* = 3 per group).

## 4. Discussion

TBI is a major health care problem worldwide. Approximately 1.5 million new cases occur annually in the United States, with mortality rates ranging from 35% to 40% in severe patients [15]. Therefore, identifying new therapeutic methods that can be used to treat TBI is essential. VPA is a simple branched-chain fatty acid with well-established efficacy for seizures [16]. It is also commonly prescribed for bipolar disorder, acute mania, and migraines [7]. The therapeutic concentration of VPA is 40 to 100 mg/mL. This therapeutic concentration is achieved using a loading dose (as low as 10 mg/kg) followed by maintenance doses (as high as 60 mg/kg). Studies have reported the HDAC inhibitory effects of 300 mg/kg or 400 mg/kg of VPA in animals [8–10]. Postinjury administration of VPA can decrease BBB permeability, reduce neural damage, and improve neurobehavioral outcomes [9]. A 400 mg/kg IP dose of VPA increased histone acetylation and reduced the activity of GSK-3 in the hippocampus [9]. When 400 mg/kg of VPA was administered 30 min after injury, it improved BBB permeability. The same dose also reduced cortical contusion and hippocampal dendritic damage and improved motor function and spatial memory. Consistent with this, HDAC inhibitors can augment memory and synaptic plasticity and promote neuronal outgrowth [17].

Although 400 mg/kg of VPA is useful in a TBI rodent model, this dose is too high for humans. The teratogenicity of VPA also limits its use in women of childbearing age. In this study, we reduced the dose to 30 mg/kg of VPA and tested its effects in rats. The results indicated that treatment with 30 mg/kg of VPA in adult rats with TBIs significantly reduced the contusion volume and the skilled forelimb reaching contusion-related deficit. Histone H3 acetylation and p-ERK and p-CREB expression were also induced in the brain with single injection of VPA at different dosages. The results reflect some of the potential effects of using 30 mg/kg/d of VPA for 7 days in the treatment of TBI.

A recent behavioral study showed that the release, collection, and manipulation hand-shaping movements involved in skilled reaching are similar in rats and humans [18]. Because hand movement plays a critical role in the quality of life of TBI patients, we conducted fine motion evaluations of skilled forelimb reaching before and after TBIs. Once the preferred reaching limb was determined, a CCI injury was performed on the contralateral motor cortex. In our study, VPA treatment reduced the contusion volume of the injured brain and improved skilled reaching motions from 14 to 28 days after a TBI. VPA might offer protection from TBIs by increasing histone acetylation and enhancing the expression of genes involved in neuronal plasticity and survival. Consistent with this, Shein et al. showed that acute treatment of TBI mice with HDAC inhibitor ITF2357 reduced contusion volume and improved motor function [19]. Zhang et al. indicated that DMA-PB (a novel HDAC inhibitor) attenuated the TBI-associated decrease in histone acetylation and reduced microglia-mediated inflammation [20]. VPA can also elicit neuronal growth by activating p-ERK [21]. Dash et al. proved that activating p-ERK after a TBI is neuroprotective. Inhibition of p-ERK exacerbates TBI-associated motor and cognitive deficits [22]. Our findings are consistent with the results of other studies [20–22] and we hypothesize that VPA plays a critical role in the neuroprotective mechanism.

The generalizability of this study is limited, because extrapolating conclusions from experiments on animal models to humans requires safety and efficacy validation. Although VPA exhibits a potential neuroprotective effect, its molecular mechanisms remain unclear. Our data indicated that 30 mg/kg of VPA treatments in rats can reduce TBI-mediated inflammation and apoptosis. However, we did not measure the kinetics of VPA in rats. Furthermore, the animal dose used (30 mg/kg) cannot be extrapolated to a human-equivalent dose. Therefore, more pharmacokinetic studies of VPA should be conducted.

In conclusion, this study identified VPA as a rational therapeutic choice for drugs aimed at treating TBI.
